# Increased Risk for Respiratory Complications in Male Extremely Preterm Infants: A Propensity Score Matching Study

**DOI:** 10.3389/fendo.2022.823707

**Published:** 2022-05-12

**Authors:** Zhiwen Su, Lili Lin, Xi Fan, Chunhong Jia, Bijun Shi, Xiaoxia Huang, Jianwei Wei, Qiliang Cui, Fan Wu

**Affiliations:** ^1^ Department of Pediatrics, The Third Affiliated Hospital of Guangzhou Medical University, Guangzhou, China; ^2^ Guangdong Provincial Key Laboratory of Major Obstetric Diseases, The Third Affiliated Hospital of Guangzhou Medical University, Guangzhou, China

**Keywords:** sex differences, extremely premature infants, outcomes, respiratory complications, propensity score matching

## Abstract

**Background:**

Many factors can affect the clinical outcome of extremely premature infants (EPIs), but the effect of sex is paradoxical. This study used propensity score matching to adjust baseline information to reassess the clinical outcome of EPIs based on sex.

**Methods:**

A retrospective analysis was performed on EPIs admitted in the Department of Neonatology of the Third Affiliated Hospital of Guangzhou Medical University from 2011 to 2020. A propensity score matching (PSM) analysis was used to adjust the confounding factors including gestational age, birth weight, 1-minute Apgar score ≤ 3, withholding or withdrawing life-sustaining treatment(WWLST), mechanical ventilation, duration of mechanical ventilation, the mother with advanced age (≥35 years old), complete-course antenatal steroid therapy and hypertensive disorders of pregnancy. The survival rate at discharge and the incidence of major complications were evaluated between the male and female groups.

**Results:**

A total of 439 EPIs were included, and 240 (54.7%) infants were males. After matching the nine confounding factors, 148 pairs of infants were finally enrolled. There was no significant difference in the survival rate at discharge, as well as the mortality of activating treatment or WWLST between the two groups (all *P*>0.05). However, the incidence of respiratory distress syndrome, bronchopulmonary dysplasia (BPD), and moderate to severe BPD in the male group was significantly increased (all *P*<0.01), especially at birth weight between 750 and 999 grams.

**Conclusions:**

The male EPIs have a higher risk of respiratory complications than females, particularly at 750 to 999 grams of birth weight.

## Introduction

Gestational age (GA), birth weight (BW), antenatal steroids administration, active resuscitation, maternal age, and hypertensive disorders of pregnancy are considered to impact the outcomes of preterm infants (1–6). There is increasing interest in perinatal medicine as to how sex impacts survival and complications of premature infants. In 1971, Naeye et al. firstly reported that male neonates had a higher risk of death (7). Over the past decades, some other clinical cohort studies have repeatedly shown that male premature infants have disadvantages in outcomes, especially in respiratory distress syndrome (RDS), bronchopulmonary dysplasia (BPD), intraventricular hemorrhage (IVH), and retinopathy of prematurity (ROP) (8–16).

However, the male disadvantage appears to be uncertain in extremely preterm infants (EPIs, defined as GA<28 weeks) or/and very preterm infants (VPIs, defined as GA<32 weeks). Binet et al. found that there was no significant difference in survival rate between male and female EPI (15). And similar research also indicated that male infants with GA below 30 weeks had comparable mortality when compared with females (16). Differently, some studies showed that male VPIs had significantly higher mortality rate (9, 11, 13, 14). BPD is regarded as the major complication in EPIs/VPIs. Kent et al. found that there was no sex difference in the incidence of BPD in infants with <29 weeks’ gestation (9). But the other studies from five national neonatal networks suggested that male EPIs/VPIs had a higher risk of BPD (11, 13–16). Similarly, some studies demonstrated that male EPIs/VPIs had a significantly higher risk of severe IVH (9, 13, 14, 16), while the study from Canadian Neonatal Network found no difference (15).

The main reason for the above conflicting results may be relative to the inconsistent baselines of the study subjects. Some characteristics such as GA, BW, and antenatal steroids, are quite different between the male and female groups, which have important impacts on mortality and morbidity in EPIs/VPIs. So, in this study, the propensity score matching method was used to adjust the baseline characteristics to estimate the impact of sex on the clinical outcome of EPIs. Furthermore, given that GA and BW were the most important factors influencing EPIs outcomes, we performed subgroup analyses of sex differences according to GA and BW classification.

## Materials and Methods

### Subjects and Data Collection

The EPIs who were admitted to the Department of Neonatology of the Third Affiliated Hospital of Guangzhou Medical University from January 1, 2011, to December 31, 2020, were selected as the research subjects. The exclusion criteria were as follows: (1) incomplete data of the infant or mother; (2) various serious congenital malformations, such as genetic metabolic diseases, central nervous system malformations, and cardiovascular malformations. The selected EPIs were divided into male and female groups, and then stratified by GA and BW, namely gestational age <26 weeks, 26 weeks, and 27 weeks, birth weight <750g, 750-999g, and ≥1000g. This study was approved by the Medical Ethics Committee of the Third Affiliated Hospital of Guangzhou Medical University. The following neonatal and maternal data were collected: (1) neonatal data: sex, GA, BW, twin/multiple pregnancy, *in vitro* fertilization (IVF), delivery method, 1- and 5-minute Apgar score, mechanical ventilation, the survival rate at discharge, and the major complications during hospitalization; (2) maternal data: age at delivery, antenatal steroid administration, and the major pregnancy complications.

### Definitions and Classifications

Survivors were defined as EPIs who survived at discharge (17). Neonatal respiratory distress syndrome (RDS) was diagnosed in preterm infants with respiratory distress shortly after birth and/or a compatible chest X-ray appearance (18). The definition and severity classification of BPD was based on the criteria established by the National Institute of Child Health and Human Development (NICHD, 2001), which was defined as oxygen dependency for at least 28 days and the severity classifications were assessed at 36 weeks postmenstrual age (PMA) or at discharge (19). The diagnosis and grading of necrotizing enterocolitis (NEC) were based on Bell’s stage (20). Retinopathy of prematurity (ROP) and its grades were defined by the international classification of ROP (21). Both IVH and periventricular leukomalacia (PVL) were diagnosed by cranial ultrasonography or magnetic resonance imaging (MRI). The Papile criterion was used to grade IVH (22), and grade III-IV IVH is served as severe IVH. PVL was defined as degeneration of white matter adjacent to the cerebral ventricles following cerebral hypoxia or brain ischemia (23). The diagnostic criteria of hospital acquired infection (HAI) referred to the diagnostic criteria of hospital infection of the Ministry of Health of China (24). In this study, HAI was limited to the infections that occurred after 48 hours of hospitalization. Treated patent ductus arteriosus (PDA) was defined as treatment with ibuprofen and/or surgical ligation after the diagnosis of PDA by echocardiography (25). Complete-course antenatal steroid therapy was defined as the administration of intramuscular injections of 6 mg dexamethasone (1 dose per 12 hours, 4 doses in total) or intramuscular injections of 12 mg betamethasone (1 dose per 12 hours, 2 doses in total) within 7 days before delivery (26, 27).

### Statistical Analysis

All data were analyzed using SPSS 26.0 software (IBM, Armonk, NY, USA). We initiated a 1:1 matched analysis by PSM with a nearest-neighbor matching algorithm to adjust the baseline characteristic differences between the two groups, including GA, BW, 1-minute Apgar score ≤ 3, WWLST, mechanical ventilation, duration of mechanical ventilation, the mother with advanced age (≥35 years old), complete-course antenatal steroid therapy and hypertensive disorders of pregnancy. These covariates were selected based on the reported studies and our data, which were found to be relative to the mortality and morbidity of preterm infants (1–5, 28–30). We used calipers of width equal to 0.02 of the standard deviation of the logit of the propensity score. Continuous variables are presented as the means ± standard deviation (SD) or as medians (P25, P75) when their distributions were highly skewed or not, and were analyzed using t-tests, or Mann–Whitney tests. Categorical variables are presented as rates, which were analyzed using Pearson chi-square tests, continuity correction chi-square tests or Fisher’s exact tests. *P*<0.05 was considered statistically significant.

## Results

### Baseline Characteristics of Neonates and Mothers

From 1 Jan 2011 to 31 Dec 2020, there were 445 EPI admitted to the Department of Neonatology, the Third Affiliated Hospital of Guangzhou Medical University. 3(0.67%), 1(0.22%), and 2(0.45%) infants were excluded due to incomplete data, genetic metabolic diseases, and the presence of congenital anomalies, respectively. At last, a total of 439 EPIs were enrolled and were divided into male or female groups. Before matching, there were 240 male infants and 199 female infants. After matching, each group included 148 infants. The baseline characteristics of the two groups before and after matching are shown in **Table 1**. Before matching, eight covariates were significantly different between the two groups, including BW, 1-minute Apgar score ≤ 3, WWLST, mechanical ventilation, duration of mechanical ventilation, the mother with advanced age (≥35 years old), complete-course antenatal steroid therapy, and hypertensive disorders of pregnancy. After matching, 296 infants were included in the PSM model. All covariates were well balanced without significant differences. The covariates of the subgroups were also well matched (**Supplemental Tables 1–3**).

**Table 1:** The characteristics of the EPIs and their mothers.

	Before matching			After matching		
	Male (No. = 240)	Female (No. = 199)	*P* value	Male (No. = 148)	Female (No. = 148)	*P* value
**Neonatal characteristic**						
Gestational age, weeks	26.7 (26.0, 27.3)	26.9 (26.1, 27.4)	0.115	26.9 (26.0, 27.4)	26.7 (26.0, 27.4)	0.916
Birth weight, g	918 (810, 1000)	870 (760, 950)	<0.01	920 (810, 980)	883 (783, 984)	0.430
WWLST, No. (%)	83 (34.6)	90 (45.2)	0.023	63 (42.6)	66 (44.6)	0.725
Apgar score, No. (%)						
1-min ≤3	26 (10.8)	10 (5.0)	0.027	9 (6.1)	9 (6.1)	1.000
1-min 4~7	81 (33.8)	67 (33.7)	0.986	52 (35.1)	46 (31.1)	0.459
5-min ≤7	42 (17.5)	28 (14.1)	0.329	25 (16.9)	20 (13.5)	0.418
Mechanical ventilation, No. (%)	200 (83.3)	140 (70.4)	<0.01	119 (80.4)	109 (73.6)	0.167
Duration of mechanical ventilation, days	4.8 (0.7, 13.5)	2.7 (0.0, 9.1)	<0.01	4.3 (0.5, 12.1)	3.0 (0.0, 10.0)	0.088
**Maternal characteristic**						
Age≥35 years old, No. (%)	80 (33.3)	46 (23.1)	0.018	42 (28.4)	37 (25.0)	0.511
Cesarean section, No. (%)	79 (32.9)	71 (35.7)	0.544	49 (33.1)	41 (27.7)	0.312
IVF, No. (%)	95 (39.6)	85 (42.7)	0.507	57 (38.5)	65 (43.9)	0.345
Twin/multiple pregnancy, No. (%)	63 (26.3)	66 (33.2)	0.113	47 (31.8)	56 (37.8)	0.272
Any antenatal steroids, No. (%)	155 (64.6)	136 (68.3)	0.407	105 (70.9)	94 (63.5)	0.173
Complete-course antenatal steroids, No. (%)	69 (28.7)	84 (42.2)	<0.01	60 (40.5)	51 (34.5)	0.280
Placental abruption, No. (%)	24 (10.0)	18 (9.0)	0.735	13 (8.8)	13 (8.8)	1.000
Placenta previa, No. (%)	16 (6.7)	12 (6.0)	0.786	8 (5.4)	10 (6.8)	0.627
Premature rupture of membranes, No. (%)	82 (34.2)	64 (32.2)	0.657	59 (39.9)	52 (35.1)	0.401
Chorioamnionitis, No. (%)	20 (8.3)	20 (10.1)	0.534	13 (8.8)	13 (8.8)	1.000
Intrauterine distress, No. (%)	9 (3.8)	7 (3.5)	0.897	8 (5.4)	5 (3.4)	0.395
Hypertensive disorder of pregnancy, No. (%)	27 (11.3)	38 (19.1)	0.021	19 (12.8)	17 (11.5)	0.722
Gestational diabetes mellitus, No. (%)	42 (17.5)	35 (17.6)	0.981	23 (15.5)	28 (18.9)	0.442
Cervical incompetence, No. (%)	43 (17.9)	34 (17.1)	0.820	30 (20.3)	25 (16.9)	0.455

Data are presented as median (IQR), or number (%); EPIs, extremely preterm infants; IVF, in vitro fertilization; WWLST, withholding or withdrawing life-sustaining treatment.

### Clinical Outcomes

There was no significant difference in the survival rate at discharge, as well as the mortality of activating treatment or WWLST between the two groups after matching (all *P*>0.05). The incidence of RDS and BPD in the male group was significantly higher than that in the female group before and after matching (all *P*<0.05). But the difference in the incidence of moderate to severe BPD between the two groups was just found after matching (48.7%[38/78] vs. 25.4%[18/71], *P*<0.01), not before matching (*P*>0.05). On the contrary, the incidence of IVH in the male group was significantly higher than that in the female group before matching (61.5%[115/187] vs. 48.5%[63/130], *P*<0.05), but not after matching (*P*>0.05). There was no significant difference in the incidence of severe IVH, PVL, NEC, ROP, HAI, or treated PDA between the two groups before or after matching. See **Table 2** for details.

**Table 2:** The clinical outcomes of the EPIs (%[diagnosed No./assessed No.]).

	Before matching			After matching		
	Male (No. = 240)	Female (No. = 199)	*P* value	Male (No. = 148)	Female (No. = 148)	*P* value
**Major morbidity**						
RDS	96.7 (232/240)	92.5 (184/199)	0.049	97.3 (144/148)	91.2 (135/148)	0.046^a^
BPD	94.8 (128/135)	83.5 (81/97)	<0.01	97.4 (76/78)	83.1 (59/71)	<0.01^a^
Moderate to severe BPD	39.3 (53/135)	28.9 (28/97)	0.101	48.7 (38/78)	25.4 (18/71)	<0.01
IVH	61.5 (115/187)	48.5 (63/130)	0.021	61.4 (70/114)	49.5 (48/97)	0.082
Severe IVH	26.2 (49/187)	18.5 (24/130)	0.107	23.7 (27/114)	19.6 (19/97)	0.473
PVL	11.8 (22/187)	5.4 (7/130)	0.053	8.8 (10/114)	5.2 (5/97)	0.308
NEC	11.3 (22/194)	7.6 (11/144)	0.257	11.5 (15/130)	4.8 (6/124)	0.053
ROP	82.2 (111/135)	80.6 (79/98)	0.754	81.8 (63/77)	78.3 (54/69)	0.591
HAI	44.1 (86/195)	48.0 (71/148)	0.476	39.8 (47/118)	49.5 (53/107)	0.144
Treated PDA	30.3 (66/218)	26.4 (43/163)	0.405	26.9 (35/130)	27.4 (34/124)	0.929
**Survival at discharge**	55.4 (133/240)	46.7 (93/199)	0.070	52.0 (77/148)	44.6 (66/148)	0.201
**Death**						
Active treatment	11.3 (27/240)	10.6 (21/199)	0.816	7.4 (11/148)	12.2 (18/148)	0.171
WWLST	33.3 (80/240)	42.7 (85/199)	0.043	40.5 (60/148)	43.2 (64/148)	0.637

a Continuity correction Chi-square tests;EPIs, extremely preterm infants; RDS, respiratory distress syndrome; BPD, bronchopulmonary dysplasia; IVH, intraventricular hemorrhage; PVL, periventricular leukomalacia; NEC, necrotizing enterocolitis; ROP, retinopathy of prematurity; HAI, hospital acquired infection; PDA, patent ductus arteriosus; WWLST, withholding or withdrawing life-sustaining treatment.

In the subgroup analysis, the male infants in the subgroup with 27 weeks of gestation had an increased incidence of BPD after matching, when compared with the female infants (94.6%[35/37] vs. 76.2%[32/42], *P*<0.05); and so did the incidence of moderate to severe BPD (40.5%[15/37] vs. 14.3%[6/42], *P*<0.01). On the other hand, the male infants in the subgroup with birth weight of 750 to 999 g, had higher incidences of RDS and BDP than female infants before or after matching (all *P*<0.05). More interestingly, the higher incidence of moderate to severe BPD in male infants was just seen after matching (50.0%[25/50] vs. 28.6%[12/42], *P*<0.05), not before matching. In the other GA and BW subgroups, the incidence of RDS, BPD, and moderate to severe BPD between male and female infants were not significantly different before or after matching (all *P*>0.05), as shown in **Tables 3**, **4**. Additionally, we made a sensitivity analysis between subgroups, which demonstrated that our results of subgroups were reliable and stable. Please see the **Supplemental Tables 4, 5**.

**Table 3:** The effects of gestational age on the incidence of respiratory complications in male and female EPIs (%[diagnosed No./assessed No.]).

Gestationalage at delivery (weeks)	Before matching			After matching		
	Male (No. = 240)	Female (No. = 199)	*P* value	Male (No. = 148)	Female (No. = 148)	*P* value
**RDS**						
<26	96.6 (56/58)	100 (38/38)	0.517^a^	100.0 (32/32)	100.0 (32/32)	–
26	97.7 (86/88)	87.7 (57/65)	0.031^b^	96.2 (51/53)	86.3 (44/51)	0.145^b^
27	95.7 (90/94)	92.7 (89/96)	0.558^b^	96.8 (61/63)	90.8 (59/65)	0.294^b^
**BPD**						
<26	100.0 (21/21)	90.9 (10/11)	0.344^a^	100.0 (11/11)	90.0 (9/10)	0.476^a^
26	98.1 (53/54)	96.0 (24/25)	1.000^b^	100.0 (30/30)	94.7 (18/19)	0.388^a^
27	90.0 (54/60)	77.0 (47/61)	0.055	94.6 (35/37)	76.2 (32/42)	<0.05^b^
**Moderate to severe BPD**						
<26	38.1 (8/21)	27.3 (3/11)	0.826^b^	36.4 (4/11)	20.0 (2/10)	0.635^a^
26	44.4 (24/54)	48.0 (12/25)	0.768	63.3 (19/30)	52.6 (10/19)	0.458
27	35.0 (21/60)	21.3 (13/61)	0.094	40.5 (15/37)	14.3 (6/42)	<0.01

^a^ Fisher’s exact tests; ^b^ Continuity correction Chi-square tests; EPIs, extremely preterm infants; RDS, respiratory distress syndrome; BPD, bronchopulmonary dysplasia.

**Table 4:** The effects of birth weight on the incidence of respiratory complications in male and female EPIs (%[diagnosed No./assessed No.]).

Birth weight at delivery (g)	Before matching			After matching		
	Male (No. = 240)	Female (No. = 199)	*P* value	Male (No. = 148)	Female (No. = 148)	*P* value
**RDS**						
<750	97.0 (32/33)	97.8 (44/45)	1.000^a^	96.0 (24/25)	100.0 (24/24)	1.000^b^
750~999	97.9 (143/146)	91.7 (110/120)	0.038^a^	99.0 (95/96)	90.1 (82/91)	0.018^a^
≥1000	93.4 (57/61)	88.2 (30/34)	0.624^a^	92.6 (25/27)	87.9 (29/33)	0.863^a^
**BPD**						
<750	100.0 (9/9)	87.5 (14/16)	0.520^b^	100.0 (8/8)	83.3 (5/6)	0.429^b^
750~999	98.7 (77/78)	84.2 (48/57)	<0.01^a^	98.0 (49/50)	83.3 (35/42)	0.034^a^
≥1000	87.5 (42/48)	79.2 (19/24)	0.354	95.0 (19/20)	82.6 (19/23)	0.431^a^
**Moderate to severe BPD**						
<750	55.6 (5/9)	31.3 (5/16)	0.397^b^	62.5 (5/8)	16.7 (1/6)	0.138^b^
750~999	43.6 (34/78)	31.6 (18/57)	0.157	50.0 (25/50)	28.6 (12/42)	0.037
≥1000	29.2 (14/48)	20.8 (5/24)	0.449	40.0 (8/20)	21.7 (5/23)	0.193

^a^ Continuity correction Chi-square tests; ^b^ Fisher’s exact tests; EPIs, extremely preterm infants; RDS, respiratory distress syndrome; BPD, bronchopulmonary dysplasia.

## Discussion

In the present study, it was clearly identified that sex played a key role in clinical outcomes of EPIs through propensity score matching analysis. Specifically, comparing with females, increasing incidences of RDS, BPD, and moderate to severe BPD existed in the male group, especially at birth weight of 750 to 999 grams.

RDS is the most common complication of EPIs. Similar to the results of previous studies (11, 13), the male EPIs was found to have a higher incidence of RDS than the female in our study. Though the exact pathogenesis remains unknown, previous studies showed that it may partly attribute to the sex differences in lung tissue structure, pulmonary surfactant, and regulation of lung development by sex hormones. The sodium transport channel in the alveolar epithelium, which is found to have lower expression in male neonates than the females, can mediate the clearance of alveolar fluid. When the fluid accumulates in the lungs by reduced clearance, it can hinder gas exchange and increase the risk of RDS (31, 32). Insufficient pulmonary surfactant is the main cause of RDS. Sex can affect the development of lung as females produce surfactants earlier than males (33, 34). Besides, the various components of pulmonary surfactants are relative to GA and sex. At 25 weeks of gestation, the proportion of dipalmitoyl phosphatidylcholine in males was lower than in females; while at 30 to 40 weeks of gestation, the concentration of saturated phosphatidylcholine and the proportion of unsaturated phosphatidylcholine in males were also lower than in females (34, 35). Sex hormones are also key to lung development. Increased concentrations of estrogen in female can activate the estrogen beta receptors, upregulate the platelet-derived growth factor alpha and enhance the granulocyte-macrophage colony stimulating factor, thereby promoting lung development and the lung surfactants secretion (36–38). Androgens can inhibit lung maturation and the production of pulmonary surfactant by downregulating epidermal growth factor and upregulating transforming growth factor β1 (38, 39).

BPD is another serious complication of EPIs. There are various risk factors for BPD, including prematurity, low birth weight, mechanical ventilation, etc. (40). Recent studies have shown that the risk factors for moderate to severe BPD in EPI include birth weight, sex, duration of mechanical ventilation, and PDA treatment (41, 42). In addition, maternal conditions such as antenatal steroid administration and hypertensive disorders of pregnancy also have impacts on severe BPD (43, 44). After matching the confounding covariates in baseline characteristics of the two groups, such as GA, BW, duration of mechanical ventilation, complete-course antenatal steroid therapy, and hypertensive disorders of pregnancy, our study found that the male EPIs had a higher incidence of BPD and moderate to severe BPD. It indicates that sex can also influence the occurrence and severity of BPD in EPIs.

Moreover, after matching, subgroup analysis found that the incidences of BPD and moderate to severe BPD in male infants with 27 weeks of gestation were higher than in females. This was similar to the report by Binet et al. (15). The sex difference in BPD incidence of EPIs with 27 weeks of gestation may be related to androgen secretion. The peak testosterone secrets at the saccular stage (26-36 weeks of gestation) (38), which has a negative impact on lung development and maturation, and the development of BPD is also at this stage. In addition, our study also demonstrated that in the subgroup of birth weight between 750 and 999 grams, the incidence of major respiratory complications in males was significantly higher than that in females, especially the incidence of moderate-to-severe BPD, while there was no significant difference in other birth weight subgroups. This may be related to the following aspects. Male infants always have a lower gestational age and are less mature than female infants with similar birth weight (15, 45). In EPIs, the benefits of each 100 g increase in birth weight are like a 1-week increase in gestational age (46).

IVH is the most common brain injury in EPIs. Antenatal steroid treatment and hypertensive disorders of pregnancy can reduce the incidence of severe IVH in EPIs (3, 5, 27, 47, 48). Our results showed that there were no significant differences in the incidence of IVH and severe IVH between the two groups after matching. This finding is consistent with the study of Shim et al. (16). Shim et al. demonstrated that male infants below 30 weeks of gestation had a higher risk for severe IVH, but after adjusting for some perinatal risk factors such as antenatal steroid therapy and hypertensive disorders of pregnancy, sex difference was not found in severe IVH in infants below 25 weeks of gestation.

To date, the sex on the survival rate of EPIs/VPIs has been contradictory (11, 13–16). The study from Canadian Neonatal Network found that there was no sex difference in survival rate at discharge in EPIs (15). Shim et al. (16) also detected that the mortality of male EPIs was near to the female, even after adjusting for perinatal risk factors such as antenatal steroids and hypertensive disorders of pregnancy. Our study found that sex was not an important impact factor on the survival rate of EPIs after matching. But it should be explained carefully, because in our study, there was a high proportion of deaths after WWLST, which could affect the survival rate analysis.

The highlight of our study is to use the propensity score matching method to unify the baseline characteristics of the two groups, and further confirm the role of sex on the major respiratory complications such as RSD, BPD, and moderate to severe BPD of EPI. However, there are still some limitations in our study. This is a single-center study, and the sample size of the subgroup is small. And it is not a randomized prospective controlled study. Before making a definite conclusion, further or multicenter studies are needed.

In conclusion, male EPIs has a higher risk of respiratory complications than female, particularly at birth weight ranging from 750 to 999 grams.

## Data Availability Statement

The original contributions presented in the study are included in the article/**Supplementary Material**. Further inquiries can be directed to the corresponding author.

## Ethics Statement

This study was approved by the Medical Ethics Committee of the Third Affiliated Hospital of Guangzhou Medical University.

## Author Contributions

QC and FW: study concept and design. ZS and LL : drafing of the manuscript. LL, XF, XH, and JW: data collection. ZS and BS: statistical analyses. FW, CJ, and ZS: review and editing. ZS, LL, and FW: Funding acquisition. All authors contributed to the article and approved the submitted version.

## Funding

This work was supported by the Natural Science Foundation of Guangdong Province (No.2021A1515011225, to FW), Guangzhou Science and Technology Project (No.202102010080, to FW), Medical Science and Technology Research Fund Project of Guangdong Province (No.A2019069, to LL), and Guangdong Science and Technology Plan Project (No. 2017ZC0252, to ZS).

## Conflict of Interest

The authors declare that the research was conducted in the absence of any commercial or financial relationships that could be construed as a potential conflict of interest.

## Publisher’s Note

All claims expressed in this article are solely those of the authors and do not necessarily represent those of their affiliated organizations, or those of the publisher, the editors and the reviewers. Any product that may be evaluated in this article, or claim that may be made by its manufacturer, is not guaranteed or endorsed by the publisher.
