# Supplementary material for: Increased Risk for Respiratory Complications in Male Extremely Preterm Infants: A Propensity Score Matching Study
Source: Front Endocrinol (Lausanne). 2022 May 12;13:823707. doi: 10.3389/fendo.2022.823707 (PMC9134850; doi:10.3389/fendo.2022.823707)
Supplement: Supplementary file 1 [file DataSheet_1.pdf]

Supplemental information

Table 1　The Characteristics of BPD in EPIs born at ≥27 weeks of gestation after matching

Characteristic	Male（No.=37）	Female（No.=42）	*P* value
**Neonatal characteristic**			
Gestational age, weeks	27.6(27.1，27.7）	27.6（27.1，27.7）	0.889
Birth weight, g	992±173	994±127	0.973
WWLST, No. (%)	1（2.7）	1（2.4）	1.000[a]
Apgar score, No. (%)			
1-min ≤3	1（2.7）	2（4.8）	1.000[a]
1-min 4～7	9（24.3）	10（23.8）	0.957
5-min≤7	5（13.5）	2（4.8）	0.332[a]
Mechanical ventilation, No. (%)	31（83.8）	32（76.2）	0.402
Duration of mechanical ventilation , days	7.3（3.0，14.7）	4.4（0.2，7.2）	0.050
**Maternal characteristic**			
Age≥35 years, No. (%)	10（27.0）	11（26.2）	0.933
Cesarean section, No. (%)	16（43.2）	16（38.1）	0.642
IVF, No. (%)	7（18.9）	13（31.0）	0.220
Twin/multiple pregnancy, No. (%)	8（21.6）	9（21.4）	0.983
Any antenatal steroids,No. (%)	31（83.8）	32（76.2）	0.402
Complete-course antenatal steroids,No. (%)	21（56.8）	16（38.1）	0.097
Placental abruption,No. (%)	3（8.1）	4（9.5）	1.000[a]
Placenta previa, No. (%)	2（5.4）	2（4.8）	1.000[a]
Premature rupture of membranes,No. (%)	16（43.2）	23（54.8）	0.307
Chorioamnionitis , No. (%)	2（5.4）	5（11.9）	0.537[a]
Intrauterine distress, No. (%)	3（8.1）	3（7.1）	1.000[a]
Hypertensive disorder of pregnancy, No. (%)	6（16.2）	5（11.9）	0.581
Gestational diabetes mellitus, No. (%)	4（10.8）	9（21.4）	0.334[a]
Cervical incompetence, No. (%)	7（18.9）	5（11.9）	0.386

Data are presented as median (IQR), mean ± SD, or number (%); IVF, in vitro fertilization; [a] Continuity correction Chi-square tests; EPIs, extremely preterm infants; BPD, bronchopulmonary dysplasia; WWLST,withholding or withdrawing life-sustaining treatment .

Table 2 The Characteristics of RDS in EPIs born at 750～999g of birth weight after matching

Characteristic	Male（No.=96）	Female（No.=91）	*P* value
**Neonatal characteristic**			
Gestational age, weeks	26.9(26.1，27.3）	26.6（26.1，27.3）	0.714
Birth weight, g	915(856，950）	875（800，930）	＜0.01
WWLST, No. (%)	37（38.5）	36（39.6）	0.886
Apgar score, No. (%)			
1-min ≤3	6（6.3）	5（5.5）	0.826
1-min 4～7	29（30.2）	29（31.9）	0.806
5-min≤7	13（13.5）	12（13.2）	0.943
Mechanical ventilation, No. (%)	75（78.1）	65（71.4）	0.291
Duration of mechanical ventilation , days	3.9（0.2，12.9）	2.9（0.0，9.1）	0.236
**Maternal characteristic**			
Age≥35 years, No. (%)	24（25.0）	23（25.3）	0.965
Cesarean section, No. (%)	34（35.4）	25（27.5）	0.243
IVF, No. (%)	39（40.6）	41（45.1）	0.541
Twin/multiple pregnancy, No. (%)	31（32.3）	36（39.6）	0.300
Any antenatal steroids,No. (%)	68（70.8）	58（63.7）	0.301
Complete-course antenatal steroids,No. (%)	40（41.7）	36（39.6）	0.769
Placental abruption,No. (%)	6（6.3）	6（6.6）	0.924
Placenta previa, No. (%)	5（5.2）	7（7.7）	0.488
Premature rupture of membranes,No. (%)	36（37.5）	29（31.9）	0.419
Chorioamnionitis , No. (%)	8（8.3）	8（8.8）	0.911
Intrauterine distress, No. (%)	5（5.2）	3（3.3）	0.776[a]
Hypertensive disorder of pregnancy, No. (%)	13（13.5）	9（9.9）	0.439
Gestational diabetes mellitus, No. (%)	16（16.7）	20（22.0）	0.357
Cervical incompetence, No. (%)	17（17.7）	12（13.2）	0.393

Data are presented as median (IQR), or number (%); IVF, in vitro fertilization; [a] Continuity correction Chi-square tests;EPIs, extremely preterm infants; RDS, respiratory distress syndrome; WWLST, withholding or withdrawing life-sustaining treatment .

.

Table 3 The Characteristics of BPD in EPIs born at 750～999g of birth weight after matching

Characteristic	Male（No.=50）	Female（No.=42）	*P* value
**Neonatal characteristic**			
Gestational age, weeks	26.9(26.1，27.4）	26.9（26.1，27.6）	0.534
Birth weight, g	920(854，950）	908（800，951）	0.379
WWLST, No. (%)	1（2.0）	1（2.4）	1.000[a]
Apgar score, No. (%)			
1-min ≤3	1（2.0）	2（4.8）	0.878[a]
1-min 4～7	14（28.0）	14（33.3）	0.580
5-min≤7	3（6.0）	3（7.1）	1.000[a]
Mechanical ventilation, No. (%)	46（92.0）	35（83.3）	0.202
Duration of mechanical ventilation , days	7.7（3.1，19.0）	5.8（2.9，20.0）	0.507
**Maternal characteristic**			
Age≥35 years, No. (%)	13（26.0）	15（35.7）	0.313
Cesarean section, No. (%)	20（40.0）	14（33.3）	0.509
IVF, No. (%)	26（52.0）	19（45.2）	0.518
Twin/multiple pregnancy, No. (%)	15（30.0）	13（31.0）	0.921
Any antenatal steroids, No. (%)	38（76.0）	33（78.6）	0.770
Complete-course antenatal steroids,No. (%)	26（52.0）	18（42.9）	0.382
Placental abruption, No. (%)	2（4.0）	5（11.9）	0.303[a]
Placenta previa, No. (%)	3（6.0）	3（7.1）	1.000[a]
Premature rupture of membranes, No. (%)	16（32.0）	15（35.7）	0.707
Chorioamnionitis, No. (%)	4（8.0）	7（16.7）	0.340[a]
Intrauterine distress, No. (%)	4（8.0）	2（4.8）	0.839[a]
Hypertensive disorder of pregnancy, No. (%)	6（12.0）	5（11.9）	0.989
Gestational diabetes mellitus, No. (%)	6（12.0）	12（28.6）	0.046
Cervical incompetence, No. (%)	12（24.0）	9（21.4）	0.770

Data are presented as median (IQR), or number (%); IVF, in vitro fertilization; [a] Continuity correction Chi-square tests;EPIs, extremely preterm infants; BPD, bronchopulmonary dysplasia; WWLST,withholding or withdrawing life-sustaining treatment .

Table 4 The effects of gestational age on the incidence of respiratory complications in male and female EPIs after matching(%[diagnosed No./assessed No.])

Gestational age at delivery(weeks)	Male(No.=148)	Female(No.=148)	*P* value
**RDS**			
＜27	97.6(83/85)	91.6(76/83)	0.159[a]
⩾27	96.8(61/63)	90.8(59/65)	0.294[a]
**BPD**			
＜27	100.0(41/41)	93.1(27/29)	0.168[b]
⩾27	94.6(35/37)	76.2(32/42)	＜0.05[a]
**Moderate to severe BPD**			
＜27	56.1(23/41)	41.4(12/29)	0.225
⩾27	40.5(15/37)	14.3(6/42)	＜0.01

[a] Continuity correction Chi-square tests; [b]Fisher's exact tests; EPIs, extremely preterm infants; RDS, respiratory distress syndrome; BPD, bronchopulmonary dysplasia.

Table 5 The effects of birth weight on the incidence of respiratory complications in male and female EPIs after matching(%[diagnosed No./assessed No.])

Birth weight at delivery(g)	Male(No.=148)	Female(No.=148)	*P* value
**RDS**			
＜900	98.4(62/63)	94.7(72/76)	0.483[a]
⩾900	96.5(82/85)	87.5(63/72)	0.071[a]
**BPD**			
＜900	96.4(27/28)	88.5(23/26)	0.551[a]
⩾900	98.0(49/50)	80.0(36/45)	0.012[a]
**Moderate to severe BPD**			
＜900	57.1(16/28)	38.5(10/26)	0.170
⩾900	44.0(22/50)	17.8(8/45)	＜0.01

[a] Continuity correction Chi-square tests; EPIs, extremely preterm infants; RDS, respiratory distress syndrome; BPD, bronchopulmonary dysplasia.
